# Patient- and Health-System-Related Barriers to Treatment Adherence for Patients with Drug-Resistant Tuberculosis in the Philippines: A Mixed-Methods Study

**DOI:** 10.1155/2022/6466960

**Published:** 2022-11-19

**Authors:** Yutaka Endo, Jahn Jaramillo, Rajendra Prasad Hubraj Yadav

**Affiliations:** ^1^Johns Hopkins Bloomberg School of Public Health, Baltimore, MD, USA; ^2^Department of Public Health Sciences, University of Miami, Miami, Florida, USA; ^3^World Health Organization, Philippines Country Office, Santa Cruz, Manila, Philippines

## Abstract

**Purpose:**

In the Philippines, drug-resistant tuberculosis (DRTB) is a growing concern. Healthcare workers face challenges in retaining patients with DRTB in care. This study intends to understand their perspectives on the factors that influence patient treatment outcomes and to propose potential programmatic solutions for strengthening care services for the patients.

**Methods:**

A mixed-methods study was conducted in the Philippines between December 2017 and March 2018 to understand the major barriers for healthcare workers to provide quality care to DRTB patients across the care continuum. In the quantitative phase, healthcare workers participated in an online survey; in the qualitative phase, in-depth interviews were conducted with a select number of the survey respondents to better understand their survey responses.

**Results:**

272 healthcare workers participated in the survey, and of those, 11 were interviewed. Survey results identified economic constraints, patient perceptions of care, family-related concerns, and limited accessibility to healthcare services as the major patient-related barriers across the care continuum. Major health-system-related barriers were insufficient human resources, lack of financial and political support, and limited knowledge about DRTB by healthcare providers. Interviews revealed more elaborate, contextualized, and nuanced aspects of each of the major challenges. The elaborated patient-related barriers included expenses needed during treatment (e.g., transportation); fear of being stigmatized by family, community, or healthcare staff; worries about adverse drug reactions from medication; a lack of family support; and the location of patients' homes. The health-system-related barriers revealed through interviews included the limited capacities of facility staff to provide DRTB care due to insufficient human resources; the shortage of funds to support treatment completion (e.g., transportation allowance and food package for patients, service vehicles and mobile phone costs for outreach actions at the facility level); and discrimination by healthcare staff against patients with DRTB attributed to the staff's limited knowledge and experiences of treating the patients.

**Conclusion:**

This study identified the main barriers for DRTB facility staff in the Philippines from the perspectives of providers. Further exploration of the barriers and best practices in facilities may be useful for improving DRTB care in the Philippines.

## 1. Introduction

Tuberculosis (TB) is a major public health issue in the Philippines. Nearly one million people had active TB in 2019, which is the third highest prevalence in the world [1]. The World Health Organization (WHO) estimates that 21,000 Filipinos were affected by DRTB in 2019 [2]. DRTB treatment outcomes also remain poor in the Philippines with a treatment success rate for multidrug resistant TB (MDRTB) of less than 60% [3].

Across the care continuum (i.e., treatment initiation, treatment retention, outreach actions, and managing adverse effects from the medicine), studies conducted in the Philippines have underscored challenges to TB care services. These studies have divided barriers into patient-related barriers and health-system-barriers [4–6]. Patient-related barriers included the location of the patients' homes, limited understanding of TB treatment, out-of-pocket expenses, and loss of wages due to participating in directly observed treatment (DOT) [4–6] Health-system-related barriers included healthcare workers' heavy workloads and low motivation, a lack of resources for conducting outreach actions, and insufficient educational opportunities for both the workers and patients.

Although various barriers to accessing quality TB care have been identified from both the patient's and community's perspectives in the Philippines [7–9], the perceptions of healthcare workers have yet to be thoroughly investigated. Their insight could shed light on the challenges to providing effective DRTB care, given their daily interactions with patients and engagement with the healthcare system. Therefore, this study sought to understand the perspectives of healthcare workers on factors that impact patient treatment outcomes in the Philippines. We sought to provide potential programmatic solutions for strengthening care services for patients with DRTB.

## 2. Methods

### 2.1. Study Design, Participants, and Data Collection

A mixed methods study was conducted between December 2017 and March 2018. Quantitative data were collected from December 2017 to January 2018 using an online survey developed with a web-based tool, SurveyMonkey®. The survey was sent to healthcare workers responsible for providing services to patients with DRTB across the Philippines. Contact information was obtained from the DRTB facility staff registry provided by a nongovernmental organization (NGO), a partner of the National TB Program (NTP), charged with hiring health professionals and deploying them to DRTB referral centers throughout the country. Emails were sent to healthcare workers by the NGO's management with a separate survey link sent by the principal investigator, YE. The survey was written in English, one of the official languages in the Philippines. Only those who provided consent at the beginning of the survey were asked to complete the survey.

The survey was developed based on our prior research and TB expert consultations with WHO on barriers and facilitators to delivering quality TB care [4–9]. The survey included both closed and open-ended questions about practices, knowledge, attitudes, barriers, and recommendations regarding the treatment and management of DRTB. Topic areas included (a) treatment initiation, (b) treatment retention, (c) patient outreach actions, and (d) the management of adverse drug reactions (ADRs), which were organized across patient- and health-system levels (Appendix). Closed-ended questions asked participants about practices (e.g., Was your facility able to deliver to your patients a type of TB care at their convenience?), knowledge (e.g., Did you know a new Department of Health (DOH) policy in regard to TB care?), and attitudes (e.g., Do you agree with the new policy?) with rating scales and yes/no questions. With the open-ended questions, participants were asked to elaborate on perceived barriers and provide recommendations for enhancing DRTB care to patients. Each question did not have to be answered in order for participants to complete the survey.

Following the survey, qualitative data were collected through semistructured telephone interviews with healthcare workers. Sampling is aimed at providing representative coverage across regions and facilities in the Philippines. The interviewed workers were purposefully selected from among those who had provided complete responses to the closed and open-ended questions on the survey in order to more profoundly understand, verify, and contextualize survey responses. Hence, this study applied a two-phase mixed methods design named the Explanatory Design, which purpose is that qualitative data help explain or build upon initial quantitative results [10]. Through an iterative approach, preliminary findings from the survey informed the development of the semistructured interview guide for the interviews, which centered around asking participants to elaborate on initial quantitative findings identified through the survey (e.g. the most common barriers in practice). This design follows the follow-up explanations model, one of two variants of the Explanatory Design, in which qualitative data is used to explain preidentified quantitative results [10]. Participants were also encouraged to expand on their needs and their recommendations for enhancing the care provided to their patients. Data analysis was as described below. Participant consent was obtained prior to all survey and interview sessions. Confidentiality was ensured by protecting participants' identities. Participant information was anonymized and securely maintained within the research team.

### 2.2. Data Analysis

Survey responses to both closed and open-ended questions collected through the survey were exported from the online platform onto an MS Excel® spreadsheet. First, responses to the closed-ended questions were counted and ranked based on the numbers in each question in order to identify major knowledge gaps, attitudes, and practices related to specific TB policies and guidelines. For the responses to the open-ended questions in the survey, the lead author (YE) read through every response to become familiar with the data and identify preliminary themes emerging from the data before exporting the responses to QDA Miner Lite®, qualitative data analysis software, for further analysis. Using the query and coding functions in the software, broad categories were developed (i.e., family-related, patient-related, economic, political support, etc.) whereby YE coded each individual response, and then measured the frequency of the categories most often mentioned by respondents in order to identify the most common barriers and recommendations. To identify emerging themes, YE re-read responses that were grouped in broad categories. Major themes were organized across patient-and health system levels. Interviews were transcribed by an independent, transcription company, and transcripts were analyzed using again QDA Miner Lite®. For the purposes of this paper, only participant responses to questions pertaining to their open-ended survey responses were analyzed.

## 3. Results

### 3.1. Study Participants

**Figure 1:**
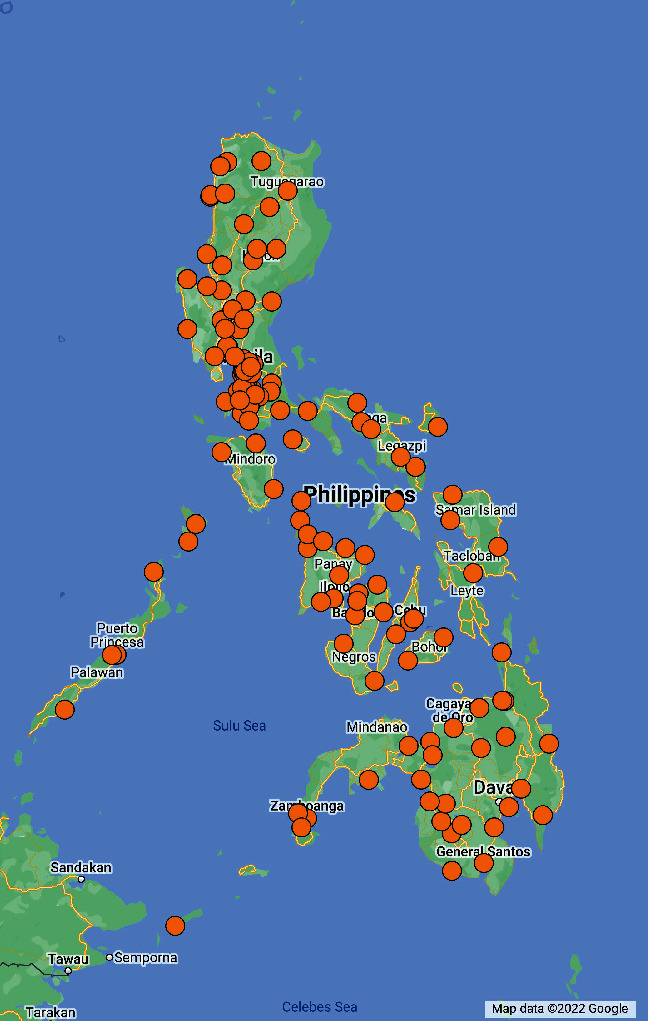
Distribution of survey participants' DRTB treatment facilities (*n* = 146).

From a total of 322 invitations sent, 272 participants from 146 DRTB treatment facilities across all 18 regions in the country completed the survey, with a response rate of 84.5% (272/322). On average, the survey took 90 minutes to complete.  Figure 1 shows the distribution of facilities by respondent. The median age range of the respondents was 26–35 years. Most respondents (82%) were nurses working in public facilities; they had worked for one to three years in the TB care field (52%).  Table 1 shows the demographic information of the respondents. Of the 272 survey respondents, a subset of 11 participants (8 nurses and 3 physicians) was interviewed (Table 2). They worked in either public or private facilities across six different regions. The interviews averaged 130 minutes per interview.

### 3.2. Main Barriers for Healthcare Workers

#### 3.2.1. Treatment Initiation

Table 3 shows the major themes identified and their associated frequencies under each topic area. A total of 252 healthcare workers responded in this topic of the survey, among which economic constraints (53%), patients' perceptions (46%), and family-related issues (27%) were the main patient-related barriers.

During the in-depth interviews, respondents further revealed that many patients could not afford treatment-related expenses, such as transportation, or stopped working to be able to complete their treatment. Many patients were the breadwinners in their households and therefore struggled with providing for their family while remaining in treatment.

*“They always prioritize what their family needs... [They] are breadwinners... [and] have to work.”* (Participant 10, nurse).

According to respondents, “patients' perceptions” specifically referred to patient concerns that prevented them from initiating treatment, such as fear of being stigmatized by their family or community, worries about ADRs during treatment or complete denial of TB-positive test results.

*“Patients… [are] afraid about* [sic] *ADRs… They can see other patients… who are already vomiting.”* (Participant 3, nurse).

When asked to expand about “family-related issues” mentioned in the survey, healthcare workers alluded to their patients who did not live with their families or those who did not have a strong support system. Limited understanding of TB by families was also related to insufficient psychosocial support provided to patients as a barrier to treatment initiation.

*“He was separated from his family… [and] prefers to drink*… *rather than the medicines.”* (Participant 8, nurse).

Based on responses from 211 participants, the most frequently mentioned health-system-related barriers included insufficient human resources (57%), inadequate financial support (29%), and poor political support (13%).

Interviewees expounded primarily on inadequate human resources. When considering the number of patients with DRTB and their other work duties, including seeing patients with other diseases, health workers felt that they did not have enough time nor the resources to provide quality care to new patients looking to begin treatment in their facilities.

*“We cater* [to] *80 patients… Daily DOT is not just our work… We do documentation, reporting, finances.”* (Participant 2, nurse).

Interviewees felt that more financial support from the government was needed, such as increasing the current transportation allowance for commuting to facilities and providing food packages to patients.

*“Some of the patients… are very far, the transportation allowance is not enough.”* (Participant 4, nurse).

Interviews clarified that the food package support was being programmatically implemented until it was discontinued in 2018, which limited the financial and political support offered to health facilities responsible for providing care to patients with TB.

#### 3.2.2. Treatment Retention

A total of 211 participants indicated that the main patient-related barriers impacting treatment retention under quality DRTB care were the perceptions of patients (28%), the location of patients' homes (18%), and a lack of family support (12%).

When asked to expand on patient perceptions, healthcare workers mentioned the difficulties to ensuring continued care of patients, particularly when symptoms waned while on treatment and patients felt “cured”, thus not feeling the need to continue treatment.

*“Some patients thought that once they feel better, they are cured of tuberculosis.”* (Participant 1, nurse).

According to interviewees, other patients who discontinued treatment felt stigmatized by other healthcare workers. The location of patients' homes was also considered a burden for patients both physically and financially, leading them to discontinue treatment.

*“During rainy seasons, they cannot go to the center… really muddy.”* (Participant 2, nurse).

Lack of family support, especially emotional and financial support, were also obstacles mentioned that affected patients' continuation and completion of treatment.

*“There is no family support… if he won't work, he won't eat.”* (Participant 6, nurse).

As compared to the aforementioned patient-related barriers, 176 respondents considered insufficient human resources (41%), facility capacity to accept decentralized patients (25%), and inadequate political support (15%) as major hindrances within the health-system.

Interviewees further explained that community treatment partners—those that treat patients at the community level and provide accessible care—were difficult to locate and provided inadequate care and treatment when the patient was decentralized. To them, the limited capacity to train treatment partners resulted from shortages in human resources.

*“Much* [sic] *more patients than the [community health workers] and sometimes it's only one nurse [at community level].”* (Participant 2, nurse).

Other healthcare workers expressed that when they referred a patient to another facility closer to the patient's residence, the facility did not accept the patient because of issues like lack of space, heavy workloads, or discrimination of patients by staff.

*“They (patients with DRTB) are… discriminated by the [referred] health center's staff.”* (Participant 1, nurse).

According to them, the discrimination experienced in these facilities where patients were referred and decentralized was due to a lack of knowledge about DRTB and infection control practices from health workers who used to deliver care for only patients with drug susceptible and thus did not have expertise in DRTB care and management. Political support from local government units for patient rehabilitation was also perceived as insufficient and considered as inadequate.

*“Maybe there are some LGUs (local government units) not to give a certain budget to health.”* (Participant 10, nurse).

#### 3.2.3. Patient Outreach Actions when Lost to Follow-Up

The main patient-related obstacles identified through 371 responses included patient adherence (31%), limited contact information (30%), and remoteness of patients' homes (23%). When attempting to reengage patients back into care, healthcare workers alluded to their experiences contacting patients who expressed anger or evaded communication with them. Some patients turned their phones off or refused to leave their homes to take directly observed treatment at health facilities according to respondents.

*“There's a patient who gets angry at us [when we conduct a home visit], ‘Why are you here? Why are you here?'”* (Participant 3, nurse).

Healthcare workers also found it challenging to carry out patient outreach actions when they could not obtain the patient's correct or complete contact information. Some patients did not have a phone, while others filled in incorrect contact information on the registry form. The distance and inaccessibility of patients' homes from DRTB treatment sites also made outreach actions challenging, given the limited time and budget required for home visits.

*“If the patient's address is really from the* [sic] *far-flung area… most of the time I do not manage to go.”* (Participant 9, nurse).

Insufficient human resources (58%), inadequate financial support (16%), and poor phone network (9%) were identified as the most frequent challenges related to the health-system, according to 316 survey responses. A shortage of healthcare workers dedicated to DRTB and heavy workloads made it difficult to conduct outreach actions such as calling and visiting patients. Healthcare workers also found it difficult to contact patients located outside of the phone network.

Additional financial and logistical support were needed by healthcare workers particularly to contact patients, according to interviewees. This is because facilities that serviced a higher number of patients who discontinued treatment received limited funds and could not afford the costs incurred from calling or texting patients from personal mobiles. Additionally, most healthcare workers did not possess dedicated service vehicles from their respective facilities. Therefore, they needed to resort to public transportation.

*“Often, we commute… [by] bus, tricycle (a three-wheeled vehicle propelled by pedals or a motor).”* (Participant 11, nurse).

#### 3.2.4. Management of ADRs including Nausea and Vomiting

According to 151 participant responses, the main patient-related challenge found regarding the management of adverse drug reactions was patient perceptions of medication (36%). Interviews suggested that some patients worried that ancillary drugs prescribed for mitigating nausea and vomiting would negatively affect their organs and worsen their health, especially if they were taking medications for other chronic conditions. This resulted in patients refusing ancillary drugs and to continue taking their treatment, according to healthcare workers.

*“There are some patients who complain of pill burden... the additional pill load.”* (Participant 5, physician).

Regarding health-system-related barriers, insufficient human resources (24%), and unavailable drugs (16%) were identified as major concerns. According to the participants that were interviewed, some ancillary drugs were not available in certain facilities, which may have reduced the likelihood of managing ADRs.

*“Some of [the ancillary] drugs are not available… not being provided by the program.”* (Participant 10, nurse).

## 4. Discussion

This study identified the main barriers to providing quality care to patients affected by DRTB in the Philippines from the perspective of frontline healthcare staff. They were identified from a wide range of views of healthcare staff collected through the distribution of a nation-wide online survey and further explored through in-depth interviews consecutively. Most of the barriers were derived from hardships that patients experienced, such as economic constraints, family-related concerns, and limited accessibility to healthcare services. On the other hand, main health-system-related challenges identified included insufficient human resources, insufficient financial and political support, and limited knowledge about DRTB by healthcare providers. Potential solutions are divided based on each primary barrier identified in the study to support health staff in the delivery of patient-centered DRTB care.

One commonly perceived barrier found in the study was the increased financial burden on patients under DRTB care, a process that takes at least nine months and can last even up to two years [11]. A 2020 patient-cost survey in the Philippines showed that 80% of patients with MDRTB faced catastrophic costs, with loss of income and transport and nutritional supplements being the largest shares [12]. These findings were confirmed by the healthcare workers in our study who expressed how challenging it was for their patients to afford daily commutes to treatment centers and other expenses for basic necessities such as food, often referencing that the transportation allowance provided by the National TB Program (NTP) of 100 Philippine pesos (approximately US$ 2) was not enough [13]. Some patients also faced loss of wages because of the need to visit health facilities for daily DOT during standard work hours.

Rather than relying on the standard rate of 100 pesos, one potential solution would be for the government to provide sufficient financial support to patients to cover loss of income and transport costs. In Niger, the NTP supported a comprehensive package for multidrug-resistant TB management, including funding sufficient to transport patients from their homes to healthcare facilities as well as nutritional support [14]. Other studies also found cash transfer programs during TB treatment to be effective in improving treatment outcomes [15, 16]. In Peru, programmatic provision of vocational training gave patients economic opportunities [17].

Limited budget at health facilities was also perceived as an obstacle for quality healthcare delivery; unavailability of facility-owned service vehicles and absence of funds for phone calls prevented healthcare workers from conducting sufficient and timely outreach activities. Given that studies emphasize the importance of adequate efforts for patient outreach actions by healthcare workers [18], adequate funding for these actions is critical [19].

Because insufficient human resources were also identified as a key administrative barrier, one possible measure to address the consequential issue of over-burdened healthcare workers is to provide basic DRTB care (e.g., DOT) at the community or home level, as indicated in the national DRTB guidelines [20] and as recommended by the WHO [21]. Other treatment administration options, including video DOT or nondaily DOT, would help reduce the workload of healthcare workers at DRTB treatment sites. Workers could then focus their attention on providing advanced DRTB care, including management of serious and unremitting ADRs. Healthcare workers at DRTB treatment facilities could play a more pronounced supervisory role and monitor patients by working closely with primary care facilities and other treatment partners. This community-based referral system would lighten the workloads of DRTB treatment sites while decreasing the physical and financial burdens on patients, which are efforts also promoted by the WHO [22]. By reducing the workload of DRTB treatment sites, they may engage primary care facilities for treatment coordination and work with them to organize community- and home-level treatment options. The NTP is already using a decentralized model of care for drug-sensitive TB patients, resulting in good treatment outcomes [23]. The same approach can be applied to patients with DRTB.

The fear of being stigmatized by the community, family, or health facility staff, as well as concerns about ADRs, were other main barriers identified by healthcare workers about why they thought patients had difficulties initiating and completing treatment. One study in Peru showed that the fear of being stigmatized among patients with DRTB was mitigated by the psychosocial support of psychiatrists and nurses [19]. In Ethiopia, TB patient support groups increased patients' confidence and reduced their fear about disclosing their condition and beginning treatment [24]. Training workshops in TB care, including education about TB, skills required for patient support, destigmatization, and human rights could help eliminate TB stigma among healthcare workers [25]. Discussions led by community volunteers and health education programs held in various community settings, such as religious buildings, have helped reduce stigma in the community [26, 27], and could be implemented in the Philippines.

Recommended measures for minimizing patients' negative feelings about ADRs include understanding patients' views about medication therapy, educating patients about the benefits of treatment, informing patients about potential ADRs and management strategies, and ensuring an updated and accurate medication list [28]. These strategies could be incorporated into a counseling curriculum that takes place throughout treatment for DRTB. Counseling with clear and accurate information about treatment has been shown to address unfavorable images about ADRs and pill burden [29]. Using oral regimens that are more effective as well as safer could potentially reduce negative experiences of patients and improve their perceptions about treatment [30].

In addition, recent studies have found that financial, physical, and emotional family support are important factors in adherence to treatment [31, 32]. Potential measures to combat this include extending educational opportunities to patients' families and strengthening family support mechanisms [15, 33]. to encourage family support, engaging family members in counseling from the beginning of the care process are recommended by the NTP in the Philippines [20]. Additional research is required to provide evidence of the role of family counseling and family-administered DOT for improving retention in care.

However, some limitations should be noted. First, there may have been response bias, and the collected responses may have been skewed towards positive responses, responses that participants only wanted to answer, or topic areas that they wanted to highlight. Since the survey was designed to permit participants to proceed throughout the survey without having to answer each question, the completeness of the data was affected. This was done to lessen the burden for healthcare workers and was mitigated through additional verification from the in-depth interviews. Next, the ambiguity of some responses to open-ended questions is noticed. However, most participants provided enough description in their answers, and only a small proportion of the open-ended answers were described with vague expressions, such as 'financial', ‘family', and ‘politic'. To avoid the risk of misinterpretation for such expressions, the authors conducted the coding process, carefully following the thematic analysis protocol and carefully considering whether to be included under a category of a particular theme or omitted from the analytic process. Additionally, the limited number of the interviewees (*n* = 11) needs to be mentioned. Although this may have had a limiting influence on fully exploring the participants' perspectives, they were carefully selected based on the completeness and comprehensiveness of responses expressed in the preliminary survey. That is expected to mitigate the risk to some extent. Lastly, to declare this study's independence of and impartiality from any influences of other individuals or organizations than the authors, the authors had full control over the data and had no agreements with funders who may have limited the study's completion. No funders had a role in the study design, data collection and analysis, decision to publish, or preparation of the manuscript.

This is the first mixed-methods study to identify major barriers impacting the initiation and retention of patients with DRTB from the perspectives of providers across the Philippines. The findings were based on the perspectives of DRTB treatment facility staff alone and excluded other actors, such as community treatment partners and patients with DRTB. However, the study explored different aspects of the treatment continuum for patients with DRTB from the perspectives of a nationally representative sample of healthcare workers.

## 5. Conclusion

This study identified the main patient- and health system-related barriers DRTB facility staff faced when providing care to patients and the factors affecting sustained medication adherence in the Philippines. The recommendations generated by this study provide guidance to the NTP program of the Philippines and advocate for improved quality care for patients affected by TB worldwide. Further exploration of the barriers, best practices in facilities, and recommendations for strengthening the NTP program from the perspective of providers could be useful for improving DRTB care in the Philippines. Evidence-based programmatic measures must be implemented to alleviate health system gaps across facilities that offer care to patients with DRTB.

## Figures and Tables

**Table 1:** Demographics of survey participants (*n* = 272).

Age	
18–25	27 (9.9%)
26–35	188 (69.1%)
36–45	35 (12.9%)
46–55	15 (5.5%)
56–65	6 (2.2%)
66–	1 (0.4%)
Gender	
Female	168 (61.8%)
Male	100 (36.8%)
Prefer not to say	4 (1.5%)
Specialty	
Physician	34 (12.5%)
Nurse	237 (87.1%)
Pharmacist	1 (0.4%)
Facility ownership type	
Public	223 (82.0%)
Private	24 (8.8%)
Nonprofit organization	20 (7.4%)
Not applicable	5 (1.8%)
Years working with TB patients	
<1	35 (12.9%)
1–3	142 (52.2%)
4–7	55 (20.2%)
8–10	17 (6.3%)
11–20	9 (3.3%)
21–	4 (1.5%)

**Table 2:** Demographics of interview participants (*n* = 11).

	Gender	Age range (years)	Profession	Experience (years)	Facility type	Region
Participant 1	Male	18-25	Project-hired nurse	1-3 years	Public	Northern
Participant 2	Female	26-35	Project-hired nurse	8-10 years	Public	Northern
Participant 3	Male	26-35	Project-hired nurse	1-3 years	Public	Northern
Participant 4	Male	26-35	Project-hired nurse	1-3 years	Public	Central
Participant 5	Male	46-55	Project-hired physician	7 years	Private	Central
Participant 6	Female	26-35	Project-hired nurse	4-7 years	Private	Central
Participant 7	Female	46-55	Permanent physician	>16 years	Public	Southern
Participant 8	Female	46-55	Project-hired nurse	11-15 years	Private	Southern
Participant 9	Male	26-35	Project-hired nurse	1-3 years	Public	Central
Participant 10	Female	26-35	Project-hired nurse	4-7 years	Public	Central
Participant 11	Female	36-45	Project-hired nurse	8-10 years	Private	Southern

**Table 3:** Major barriers, examples, and frequencies of open-ended survey responses to selected questions on major topics of DRTB care.

*Patient-related barriers*			*Health-system-related barriers*		
Themes	Reply no. (%)	Examples from open-ended survey questions	Themes	Reply no. (%)	Examples from open-ended survey questions
Topic a. Treatment initiation					
	n =252			n =211	
Economic constraints	134 (53%)	*Not able to continue work lacking money for transportation*	Human resources	120 (57%)	*Too many patients*
Patient perceptions	115 (46%)	*Fear of treatment side effects*	Financial support	61 (29%)	*Absence of food package*
Family-related issues	67 (27%)	*Lack of support from family*	Limited political support	27 (13%)	*Not supportive* [sic] *LGU*
Topic b. Treatment retention					
	*n* = 211			*n* = 176	
Patient perceptions	59 (28%)	*Stigma coming from the…worker*	Human resources	73 (41%)	*Nurse[s] [have] no time for field visits*
Location of patients' homes	38 (18%)	*Centers not capable of TB care*	Facility capacity	44 (25%)	*[PCC is] afraid of handling DRTB*
Lack of family support	26 (12%)	*No family support for food*	Limited political support	27 (15%)	*Insufficient financial support*
Topic c. Patient outreach actions					
	*n* = 371			*n* = 316	
Patient compliance	116 (31%)	*Patients refused visit*	Human resources	176 (56%)	*Because of workload, [we] neglect to contact patients*
Contact information	113 (30%)	*Patient has no phone at all*	Limited financial support	50 (16%)	*Vehicle should be provided*
Remoteness of patient homes	22 (12%)	*Unavailability of transportation*	Poor phone coverage	29 (9%)	*There is no network coverage*
Topic d. Management of nausea and vomiting					
	*n* = 151			*n* = 124	
Patient perceptions of medication	54 (36%)	*Concerned [with] additional medications*	Human resources Unavailable drugs	34 (27%) 20 (16%)	*No staff to monitor nausea* *Interrupted medicine supply*

**Notes:** Respondents' numbers in topic c (*n* = 371 for patient-related barriers, *n* = 316 for health-system-related barriers) were more than 272, the total number of participants in the survey, because responses to two different questions regarding topic c were compiled.

**Abbreviations:** ADR: adverse drug reaction; CTP: community treatment partner; DOH: Department of Health; LGU: local government unit; NTP: National Tuberculosis Control Program; PCC: primary care center, TB: tuberculosis.

## Data Availability

Used data is not publicly available due to ethical concerns as the interview audio data analyzed for this research are confidential.

## Appendix

Survey questions on major topics of DRTB care

### A. Introduction

We have designed this survey to understand your opinions and the challenges that you face during your day-to-day work. In addition, we would like to hear your ideas on how we can support you to do your work better. Hence, please feel free to provide your constructive ideas openly and honestly.

### B. General Information

1. What is your sex?

   ( ) Male

   ( ) Female

   ( ) Prefer not to say

(2) What is your age group?

   ( ) <18 years old

   ( ) 18-25 years old

   ( ) 26-35 years old

   ( ) 36-45 years old

   ( ) 46-55 years old

   ( ) 56-64 years old

   ( ) >65 years old

   ( ) Others (please specify)

(3) What is your profession?

   ( ) Physician

   ( ) Nurse

   ( ) Medical technician

   ( ) Midwife

   ( ) Others (please specify)

(4) How many years have you worked in the field of TB control?

   ( ) <6 months

   ( ) 6-12 months

   ( ) 1-3 years

   ( ) 4-7 years

   ( ) 8-10 years

   ( ) 11-15 years

   ( ) 16-20 years

   ( ) >20 years

   ( ) Others (please specify)

(5) What is full name of your multidrug resistant TB (MDRTB) center?: ____________
(6) What is the ownership type of your facility?

   ( ) Public

   ( ) Private

   ( ) Nonprofit organization

   ( ) Others (please specify)

### C. Topic a. Treatment Initiation (Survey Section Title: Turnaround Time)

1. Was your facility able to start MDRTB treatment within one week from the day of Xpert^∗^ test results for at least 90% of your patients in the past three months?

^∗^Xpert: a test with a machine named GeneXpert contributing to the rapid diagnosis of TB and drug resistance.

   ( ) Yes

   ( ) Maybe

   ( ) Maybe not

   ( ) No

(2) What are the main patient-related challenges (e.g., patient's refusal, patient's family issue) that prevented your facility from doing that?

   Challenge 1: ____________

   Challenge 2: ____________

   Challenge 3: ____________

(3) What are the main system-related challenges (e.g., manpower, finance, NTP policy) that prevented your facility from doing that?

   Challenge 1: ____________

   Challenge 2: ____________

   Challenge 3: ____________

(4) What are your recommendations to overcome those challenges?

   Recommendation 1: ____________

   Recommendation 2: ____________

   Recommendation 3: ____________

### D. Topic b. Treatment Retention (Survey Section Title: Patient Travel Time)

1. Has your facility been able to ensure that at least 90% of all your current patients (especially those who have missed four doses or more) are taking DOT at a location that is within 10 minutes of travel time from their workplace or home?

   ( ) Yes

   ( ) Maybe

   ( ) Maybe not

   ( ) No

(2) What are the main patient-related challenges (e.g., patient's refusal, patient's family issue) that prevented your facility from doing that?

   Challenge 1: ____________

   Challenge 2: ____________

   Challenge 3: ____________

(3) What are the main system-related challenges (e.g., manpower, finance, NTP policy) that prevented your facility from doing that?

   Challenge 1: ____________

   Challenge 2: ____________

   Challenge 3: ____________

(4) What are your recommendations to overcome those challenges?

   Recommendation 1: ____________

   Recommendation 2: ____________

   Recommendation 3:____________

### E. Topic c: Patient Outreach Actions (Survey Section Title: Case Holding)

1. According to the new DOH policy, if a patient misses taking a dose for the first time, the PMDT facility staff needs to send an SMS or call the patient. If the patient misses a second dose at any time during the treatment, the PMDT or the nearest health facility staff needs to visit the patient's home and provide supervised treatment along with adherence counseling of the patient and their family members.

- Was your facility able to send an SMS or call at least 90% of the patients who missed their first dose in the past three months? (This question is only for patients who are taking DOT from your facility)

   ( ) Yes

   ( ) Maybe

   ( ) Maybe not

   ( ) No

(ii) What are the main patient-related challenges (e.g., patient's refusal, patient's family issue) that prevented your facility from doing that?

   Challenge 1: ____________

   Challenge 2: ____________

   Challenge 3: ____________

(iii) What are the main system-related challenges (e.g., manpower, finance, NTP policy) that prevented your facility from doing that?

   Challenge 1: ____________

   Challenge 2:____________

   Challenge 3: ____________

(iv) What are your recommendations to overcome those challenges?

   Recommendation 1: ____________

   Recommendation 2: ____________

   Recommendation 3: ____________

(2) According to the new DOH policy, if a patient misses to take a dose for the first time, the PMDT facility staff needs to send an SMS or call the patient. If the patient misses a second dose at any time during the treatment, the PMDT or the nearest health facility staff needs to visit the patient's home and provide supervised treatment along with adherence counseling of the patient and their family members.
  - Was your facility able to visit the homes of at least 90% of the patients who missed their second dose in the past three months? (This question is only for patients who are taking DOT from your facility)

   ( ) Yes

   ( ) Maybe

   ( ) Maybe not

   ( ) No

(ii) What are the main patient-related challenges (e.g., patient's refusal, patient's family issue) that prevented your facility from doing that?

   Challenge 1: ____________

   Challenge 2: ____________

   Challenge 3: ____________

(iii) What are the main system-related challenges (e.g., manpower, finance, NTP policy) that prevented your facility from doing that?

   Challenge 1: ____________

   Challenge 2: ____________

   Challenge 3: ____________

(iv) What are your recommendations to overcome those challenges?

   Recommendation 1: ____________

   Recommendation 2: ____________

   Recommendation 3: ____________

### F. Topic d. Management of Nausea and Vomiting (Survey Section Title: Five-Step Care for Nausea/Vomiting)

According to the new DOH policy, here are the five steps to prevent or treat nausea/vomiting as an adverse drug reaction caused by any of the MDRTB drugs, especially Prothionamide and para-aminosalicylic acid (PAS): 1. Take a light meal about one hour before drug intake, 2. Divide the dose of Prothionamide into two doses to be taken at two different times each day, 3. Provide Metoclopramide about one hour before drug intake, 4. Provide Ondansetron about one hour before drug intake, 5. Decrease dose or discontinue the suspected drug.

1. If you have any patient who still has nausea/vomiting, have you tried all the five steps already for those patients in the last three months?

   ( ) Yes

   ( ) Maybe

   ( ) Maybe not

   ( ) No

(2) What are the main patient-related challenges (e.g., patient's refusal, patient's family issue) that prevented your facility from doing that?

   Challenge 1: ____________

   Challenge 2: ____________

   Challenge 3: ____________

(3) What are the main system-related challenges (e.g., manpower, finance, NTP policy) that prevented your facility from doing that?

   Challenge 1: ____________

   Challenge 2: ____________

   Challenge 3: ____________

(4) What are your recommendations to overcome those challenges?

   Recommendation 1: ____________

   Recommendation 2: ____________

   Recommendation 3: ____________

## References

[1] Gundo W., World Health Organization Representative Office for the Philippines (2019). It’s time to end TB in the Philippines. https://www.who.int/philippines/news/commentaries/detail/it-s-time-to-end-tb-in-the-philippines.

[2] World Health Organization (2021). Tuberculosis profile: Philippines. https://worldhealthorg.shinyapps.io/tb_profiles/?_inputs_&entity_type=%22country%22&lan=%22EN%22&iso2=%22PH%22.

[3] The Philippines Department of Health (2019). Philippines TB joint TB program review: Oct 3-14, 2019. *Manila, the Philippines: the Philippines Department of Health*.

[4] Hasker E., Khodjikhanov M., Sayfiddinova S. (2010). Why do tuberculosis patients default in Tashkent City, Uzbekistan? A qualitative study. *The International Journal of Tuberculosis and Lung Disease*.

[5] Kizub D., Ghali I., Sabouni R. (2012). Qualitative study of perceived causes of tuberculosis treatment default among health care workers in Morocco. *The International Journal of Tuberculosis and Lung Disease*.

[6] Negandhi H., Tiwari R., Sharma A. (2017). Rapid assessment of facilitators and barriers related to the acceptance, challenges and community perception of daily regimen for treating tuberculosis in India. *Global Health Action*.

[7] Hu A., Loo E., Winch P. J., Surkan P. J. (2012). Filipino women’s tuberculosis care seeking experience in an urban poor setting: a socioecological perspective. *Health Care Women International.*.

[8] Tupasi T. E., Garfin A. M. C. G., Kurbatova E. V. (2016). Factors associated with loss to follow-up during treatment for multidrug-resistant tuberculosis, the Philippines, 2012–2014. *Emerging Infectious Diseases*.

[9] Lagrada L. P., Uehara N., Kawahara K. (2008). Analysis of factors of treatment completion in DOTS health facilities in Metro Manila, Philippines: a case-control study. *Kekkaku (Tuberculosis).*.

[10] Creswell J. W., Clark V. L. (2017). Designing and conducting mixed methods research. *Sage Publications*.

[11] World Health Organization (2018). *Rapid communication: key changes to treatment of multidrug-and rifampicin-resistant tuberculosis (MDR/RR-TB)*.

[12] Tomeny E., Mendoza V. L., Marcelo D. B. (2020). Patient-cost survey for tuberculosis in the context of patient-pathway modelling. *The International Journal of Tuberculosis and Lung Disease*.

[13] The Philippines Department of Health (2013). Joint Tuberculosis Program Review, Philippines. *The Philippine Department of Health*.

[14] Fund G (2018). *Best Practices on TB Case Finding and Treatment: Reflections and Lessons from West and Central Africa and beyond*.

[15] Ukwaja K. N., Alobu I., Gidado M., Onazi O., Oshi D. C. (2017). Economic support intervention improves tuberculosis treatment outcomes in rural Nigeria. *The International Journal of Tuberculosis and Lung Disease*.

[16] Oliosi J. G., Reis-Santos B., Locatelli R. L. (2019). Effect of the Bolsa Familia Programme on the outcome of tuberculosis treatment: a prospective cohort study. *The Lancet Global Health*.

[17] Paz-Soldán V. A., Alban R. E., Jones C. D., Oberhelman R. A. (2013). The provision of and need for social support among adult and pediatric patients with tuberculosis in Lima, Peru: a qualitative study. *BMC Health Services Research*.

[18] Jha U. M., Satyanarayana S., Dewan P. K. (2010). Risk factors for treatment default among re-treatment tuberculosis patients in India, 2006. *PLoS One*.

[19] Acha J., Sweetland A., Guerra D., Chalco K., Castillo H., Palacios E. (2007). Psychosocial support groups for patients with multidrug-resistant tuberculosis: five years of experience. *Global Public Health*.

[20] The Philippines Department of Health (2017). *Programmatic Management of Drug-Resistant Tuberculosis (PMDT) Implementing Guidelines*.

[21] World Health Organization (2017). Guidelines for treatment of drug-susceptible tuberculosis and patient care, 2017 update. *World Health Organizations*.

[22] World Health Organization (2018). *Global tuberculosis report 2018*.

[23] Romualdez A. G. (2007). TB-DOTS in the Philippines: impact of decentralization and health sector reform. *Bulletin of the World Health Organization*.

[24] Demissie M., Getahun H., Lindtjørn B. (2003). Community tuberculosis care through "TB clubs" in rural North Ethiopia. *Social Science & Medicine*.

[25] Wu P. S., Chou P., Chang N. T., Sun W. J., Kuo H. S. (2009). Assessment of changes in knowledge and stigmatization following tuberculosis training workshops in Taiwan. *Journal of the Formosan Medical Association*.

[26] Balogun M., Sekoni A., Meloni S. T. (2015). Trained community volunteers improve tuberculosis knowledge and attitudes among adults in a periurban community in Southwest Nigeria. *The American Journal Of Tropical Medicine And Hygiene*.

[27] Croft R. P., Croft R. A. (1999). Knowledge, attitude and practice regarding leprosy and tuberculosis in Bangladesh. *Leprosy Review*.

[28] American Society of Health-System Pharmacists (1995). ASHP guidelines on adverse drug reaction monitoring and reporting. American Society of Hospital Pharmacy. *American Journal of Health-System Pharmacy*.

[29] Gebremariam M. K., Bjune G. A., Frich J. C. (2010). Barriers and facilitators of adherence to TB treatment in patients on concomitant TB and HIV treatment: a qualitative study. *BMC Public Health*.

[30] World Health Organization (2019). *WHO Consolidated Guidelines on Drug-Resistant Tuberculosis Treatment*.

[31] Gebreweld F. H., Kifle M. M., Gebremicheal F. E. (2018). Factors influencing adherence to tuberculosis treatment in Asmara, Eritrea: a qualitative study. *Journal of Health, Population and Nutrition*.

[32] Kaulagekar-Nagarkar A., Dhake D., Jha P. (2012). Perspective of tuberculosis patients on family support and care in rural Maharashtra. *The Indian Journal of Tuberculosis*.

[33] Munro S. A., Lewin S. A., Smith H. J., Engel M. E., Fretheim A., Volmink J. (2007). Patient adherence to tuberculosis treatment: a systematic review of qualitative research. *PLoS Medicine*.

